# The Origin of Sars-CoV-2: Why It Matters

**DOI:** 10.3389/fpubh.2021.719914

**Published:** 2021-09-08

**Authors:** Paolo Vineis, Stefania Salmaso

**Affiliations:** ^1^Department of Epidemiology and Biostastistics, Imperial College London, London, United Kingdom; ^2^Istituto Superiore di Sanità, Rome, Italy; ^3^Italian Society of Epidemiology, Rome, Italy

**Keywords:** SARS-CoV-2, Wuhan, bats, Mojian mine, COVID-19

As Bloom et al. (1) have indicated in a letter to the Science magazine, clarity should be made about the origins of Sars-CoV-2, outside any political out-of-context use of limited scientific evidence. The accusations toward China, and specifically the Wuhan virology laboratory, have been based so far on little evidence and much political speculation. It is time to collect evidence, before making statements based on “likelihood” that the origin of the virus is “natural” (from bats) or not (from a laboratory). This was the message in Bloom and colleagues' letter, and in the accompanying, balanced, Editorial by Thorp (2). Why is it important to know? Not only to stop political speculations on both sides, but mainly to help science to reconstruct the causal chain and be able to predict next spillovers, either from laboratory accidents or from the wildlife. At stake there is not only the safety of laboratories that manipulate living organisms, but also a better understanding of future scenarios related to natural degradation and planetary overload. We need to know the impact of deforestation, animal farming, animal markets, etc. on the risk of spillovers, to be able to prevent them.

As reported by a paper published in Frontiers in Public Health in 2020 (3), commented upon more recently by Speciale (4), 6 workers in 2012 developed severe pneumonia with symptoms very similar to those of COVID-19. They worked in a copper mine in Mojiang, in the Yunnan province (China), to clean huge guano deposits left by bats. Samples were collected from the miners and from bats, and then analyzed for viruses at the Wuhan laboratory. In October 2020, Rahalkar and Bahulikar (3) proposed that the Mojiang mineshaft miners' illness could provide important clues to the origin of SARS-CoV-2. They suggested that there were striking similarities between the Mojiang pneumonia cases and COVID-19, and reported about a Master's thesis that concluded that the pneumonia in the miners was due to a SARS-like CoV from horseshoe bats. These conclusions were contradicted by researchers at the Wuhan Institute of Virology in a Nature paper (5), which did not find evidence of SARS-CoV infection among the workers. However, in the same paper phylogenetic analysis indicated that a coronavirus collected from bats in the cave and designated RaTG13 had a 96% resemblance to SARS-CoV-2. Therefore, the case is still open.

There are several remaining questions about the Sars-CoV-2 pandemic but one of the most important is to understand how the virus that initiated the epidemic spread in Wuhan City and to identify the missing links between the viruses circulating in bat populations and the human virus. Different theories of accidental release from a lab and zoonotic spillover remain possible (1) and the currently available evidence does not allow us to draw a firm, straightforward conclusion. Part of the problem is that the controversy over the epidemic origin has become part of a political debate, while independent reliable research and evidence is needed as to whether SARS-CoV-2 came directly from bats or indirectly through intermediate hosts.

This story is only a small piece of a probably much larger puzzle that involves wildlife and laboratories. There is certainly a need to “document the veracity and provenance of data from which analyses are conducted and conclusions drawn so that analyses are reproducible by independent experts” as Bloom's letter indicates (1).

## Author Contributions

All authors listed have made a substantial, direct and intellectual contribution to the work, and approved it for publication.

## Conflict of Interest

The authors declare that the research was conducted in the absence of any commercial or financial relationships that could be construed as a potential conflict of interest.

## Publisher's Note

All claims expressed in this article are solely those of the authors and do not necessarily represent those of their affiliated organizations, or those of the publisher, the editors and the reviewers. Any product that may be evaluated in this article, or claim that may be made by its manufacturer, is not guaranteed or endorsed by the publisher.
